# Adjusting for gene-specific covariates to improve RNA-seq analysis

**DOI:** 10.1093/bioinformatics/btad498

**Published:** 2023-08-17

**Authors:** Hyeongseon Jeon, Kyu-Sang Lim, Yet Nguyen, Dan Nettleton

**Affiliations:** Department of Biomedical Informatics, The Ohio State University, Columbus, OH, United States; Pelotonia Institute for Immuno-Oncology, The James Comprehensive Cancer Center, The Ohio State University, Columbus, OH 43210, United States; Department of Animal Resources Science, Kongju National University, Yesan-gun, Chungnam 32439, Republic of Korea; Department of Mathematics and Statistics, Old Dominion University, Norfolk, VA 23529, United States; Department of Statistics, Iowa State University, Ames, IA 50011, Unites States

## Abstract

**Summary:**

This article suggests a novel positive false discovery rate (pFDR) controlling method for testing gene-specific hypotheses using a gene-specific covariate variable, such as gene length. We suppose the null probability depends on the covariate variable. In this context, we propose a rejection rule that accounts for heterogeneity among tests by using two distinct types of null probabilities. We establish a pFDR estimator for a given rejection rule by following Storey’s *q*-value framework. A condition on a type 1 error posterior probability is provided that equivalently characterizes our rejection rule. We also present a suitable procedure for selecting a tuning parameter through cross-validation that maximizes the expected number of hypotheses declared significant. A simulation study demonstrates that our method is comparable to or better than existing methods across realistic scenarios. In data analysis, we find support for our method’s premise that the null probability varies with a gene-specific covariate variable.

**Availability and implementation:**

The source code repository is publicly available at https://github.com/hsjeon1217/conditional_method.

## 1 Introduction

Gene expression refers to messenger RNA transcript abundance quantified by RNA profiling techniques. The invention of RNA-seq enables researchers to profile nearly all genes in an organism simultaneously. Research questions involving RNA-seq data often focus on identifying genes differentially expressed (DE) across different experimental conditions. Genes not DE are called equally or equivalently expressed (EE) genes. DE genes are typically identified through hypothesis testing on each gene in a statistical framework, viewed as a multiple testing problem. When dealing with gene expression data under the multiple testing framework, the most useful error quantity is typically the false discovery rate (FDR), introduced by Benjamini and Hochberg (1995). FDR refers to the expected proportion of false positives among all tests whose null hypotheses have been rejected. The most widely used procedure is Storey’s (2002)*q*-value method.

Contemporary methods for FDR control are based on gene-specific covariate variables such as mean nonzero expression and the proportion of samples with the nonzero expression (Korthauer *et al.* 2019). As circumstances vary across hypothesis tests, it is vital to consider each test separately. Cai and Sun (2009) developed an FDR-controlling method using external grouping information. An FDR regression method proposed by Scott *et al.* (2015) regulates FDR by utilizing the local FDR and treating the null probability as a function of covariate variables. Lei and Fithian (2016) and Li and Barber (2019) also used prior information regarding a specific predetermined structure in the pattern of locations of the signals and nulls within the list of hypotheses, such as ordered structure, to adjust the *P*-values adaptively. Boca and Leek (2018) also proposed a method (BL), considering the FDR and null probability as functions of a covariate variable. Ignatiadis *et al.* (2016) and Ignatiadis and Huber (2021) proposed an independent hypothesis weighting method (IHW) which maximizes the number of rejected null hypotheses, based on a covariate-variable-based group. Recently, Lei and Fithian (2018) developed a covariate-specific *P*-value thresholding method (AdaPT), based on adaptively determined significance thresholds.

The AdaPT method has developed into a powerful approach that is expected to yield more discoveries by focusing on promising hypotheses and utilizing adaptively defined *P*-value rejection thresholds. Initially, the method establishes a constant threshold across all covariate values. The initial threshold is updated continuously to gradually increase rejection power. As a result of considering multiple thresholds, we predict that the method’s average ability to classify the true positives across all nominal FDR levels may deteriorate. Simultaneously, adaptively determined thresholds complicate FDR estimation.

This article presents a novel and more straightforward rejection rule that accounts for the heterogeneity between hypotheses. Specifically, our rejection rule is based on the product of the *P*-value and covariate-specific conditional null probability, given the *P*-value is no larger than α. Due to the simplicity, the approach easily demonstrates positive FDR (pFDR) control of the type suggested by Storey (2002). Because pFDR provides an upper bound on FDR, our approach also provides FDR control. We demonstrate that the rejection rule is uniquely determined by a property of equalizing a particular conditional type 1 error posterior probability across tests.

Recently, it was discovered that there exist relationships between biological timing and gene length: shorter genes tend to regulate immediate physical processes such as skin recovery, whereas longer genes tend to regulate long-term physical processes such as muscle development (Lopes *et al.* 2021). Thus, the fraction of DE or EE genes may vary by gene length depending on the experimental conditions studied. From a Bayesian perspective, the null probability may vary by gene length. Because of this heterogeneity, we consider gene length as a covariate variable potentially important to consider when identifying DE genes. Though we focus exclusively on gene length in this article, our approach is applicable for any gene-specific covariate.

The remainder of this article is organized as follows. In Section 2, we define our method in detail and argue its mathematical implications in terms of posterior probability. In Section 3, we demonstrate the effectiveness of the method through simulation studies. In Section 4, we illustrate our method’s efficacy through data analysis. Lastly, Section 5 evaluates the proposed method’s potential for further development.

## 2 Materials and methods

Our research objective is to declare genes to be DE while controlling pFDR in the multiple testing framework. Our method is inspired by Storey’s (2002)*q*-value method based on the Bayesian perspective. Following the Bayesian perspective, we consider two types of conditional prior probabilities of being an EE gene, also referred to as conditional null probabilities. Both conditional null probabilities are considered as functions of a covariate variable. Section 2.1 presents a rejection rule based on a conditional null probability. By inverting the rejection rule, its rejection region is naturally determined in Section 2.2. In Section 2.3, we establish the pFDR estimator and *q*-value estimator based on another conditional null probability through mathematical reasoning. Section 2.4 describes a procedure for estimating the conditional null probabilities, which serves as the foundation for our method. Section 2.5 delves into the rejection rule’s intrinsic meaning regarding posterior probability.

### 2.1 Rejection rule

Our rejection rule is based on the premise that a *P*-value rejection threshold should be negatively associated with null probability. Furthermore, we assume that null probability is associated with a gene-specific covariate. This assumption is reasonable given the change in the fraction of DE genes with gene length discussed in the previous section. Therefore, we present a rejection rule based on the conditional null probability, given the covariate and an event involving the *P*-value.

Consider hypothesis testing for each of *m* genes. For gene j∈{1,…,m}, let $X_{j}$ and $P_{j}$ denote the value of a covariate and the *P*-value, respectively. Let $H_{0j}$ denote the event that gene *j* is an EE gene. Let


(1) $$\begin{array}{c} \pi_{0}(X_{j})=P(H_{0j}|X_{j})\;\text{and} \end{array}$$

(2) $$\pi_{0|\alpha }(X_{j})=P(H_{0j}|P_{j}\leq \alpha ,X_{j}).$$

Expressions (1) and (2) are conditional probabilities of gene *j* being an EE gene. These conditional null probabilities are functions of the covariate value $X_{j}$. Furthermore, (2) is the conditional null probability conditioning on the *j*th *P*-value being no larger than α. It is worth noting that α can either be specified as a value or selected via a procedure, as described in Section 3.2. Define the *j*th $\tilde{p}$-value as ${\tilde{P}}_{j}$ = $P_{j}\cdot \pi_{0|\alpha }(X_{j})$. The following is the rejection rule we propose:

**Rejection Rule 2.1**. Reject all null hypotheses whose $\tilde{p}$-value ≤ *t*, for some t>0.

The genes declared to be DE (DDE) following Rejection Rule 2.1 are naturally determined by $\left\{j:{\tilde{P}}_{j}\leq t\right\}$. Under the rejection rule, both the *P*-value and the conditional null probability in (2) affect the rejection decision for each hypothesis test. Note that we initially assume that $\pi_{0}(\cdot )$ and $\pi_{0|\alpha }(\cdot )$ are known and then replace these functions with estimates discussed in Section 2.4. Section 2.5 discusses the rejection rule’s intrinsic meaning.

### 2.2 Rejection region

By inverting the rejection rule, the rejection region for the *P*-value of the *j*th gene can be obtained as follows:


(3) $$\begin{array}{c} \Gamma_{X_{j}}(t)=\{p\in [0,1]:p\cdot \pi_{0|\alpha }(X_{j})\leq t\}=[0,u_{t}(X_{j})], \end{array}$$

where $u_{t}(X_{j})=1$ if $\pi_{0|\alpha }(X_{j})\leq t$ and $u_{t}(X_{j})=\frac{t}{\pi_{0|\alpha }(X_{j})}$ otherwise. Note that


(4) $${\tilde{P}}_{j}\leq t\Leftrightarrow P_{j}\in \Gamma_{X_{j}}(t)\Leftrightarrow P_{j}\leq u_{t}(X_{j}).$$

**Figure 1.**
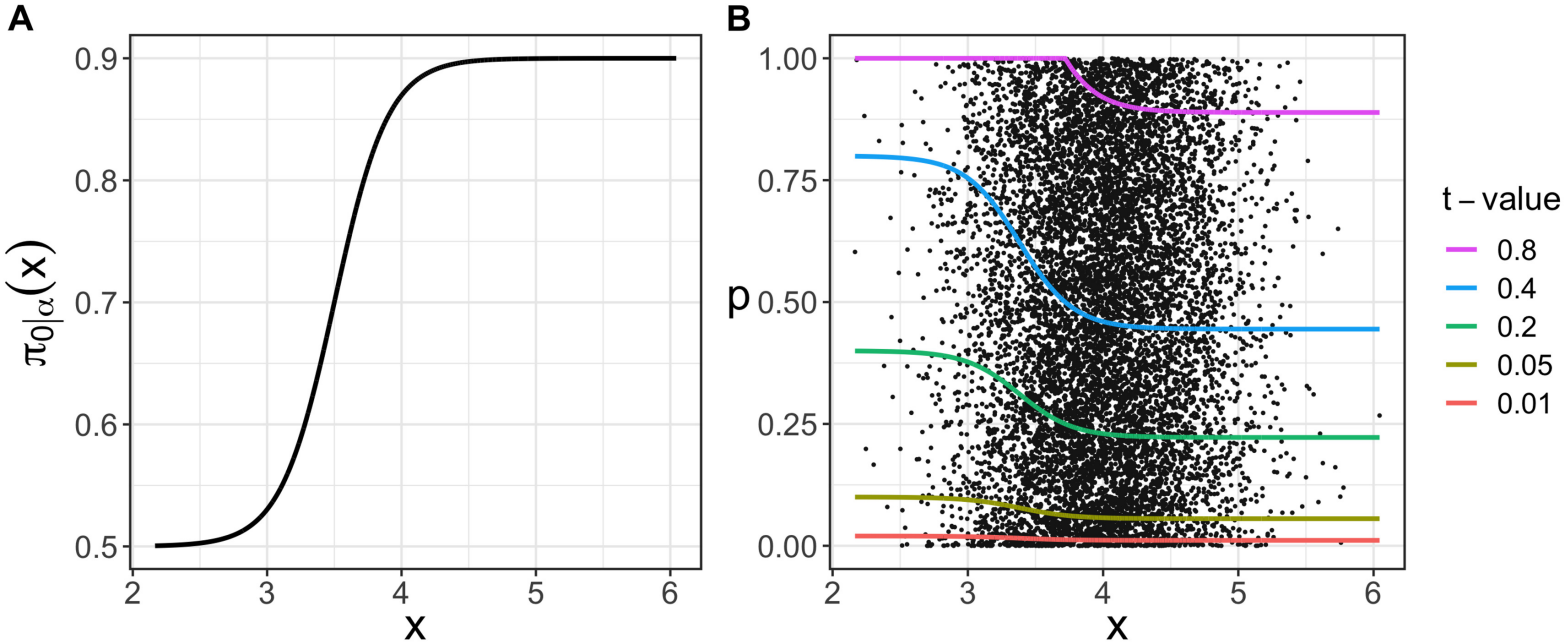
An example function $\pi_{0|\alpha }(x)$ is depicted in (A), and the rejection regions’ upper bounds created by five distinct *t*-values are illustrated in (B).

Considering the rejection region associated with a rejection rule is useful for estimating the pFDR and for gaining a better understanding of the rule. Figure 1B illustrates how the rejection region’s upper bound varies with *x* for various *t*-values for the arbitrarily chosen $\pi_{0|\alpha }(x)$ in Fig. 1A. In addition, Fig. 1B demonstrates that genes with relatively high *P*-values may, nonetheless, be declared to be DE genes when their conditional null probabilities are low. The phenomenon is noticeable when *x* is between 2 and 3.

### 2.3 False discovery rate estimator

For a given $\tilde{p}$-value significance threshold *t*, the number of genes declared to be DE is


(5) $$R(t)=\sum_{j=1}^{m}1({\tilde{P}}_{j}\leq t).$$

The number of false positives among the R(t) genes can be expressed by


(6) $$V(t)=\sum_{j=1}^{m}V_{j}(t),\text{where}\;V_{j}(t)=1({\tilde{P}}_{j}\leq t,H_{0j}).$$

From (4), $V_{j}(t)$ has another expression: pFDR can be defined as $\text{pFDR}(t)=E\{\frac{V(t)}{R(t)}|R(t)>0\}$. For a generalized significance region $\tilde{\Gamma }$ of ${\tilde{P}}_{j}$, $V(\tilde{\Gamma })$ and $R(\tilde{\Gamma })$ can be naturally defined by replacing ${\tilde{P}}_{j}\leq t$ with ${\tilde{P}}_{j}\in \tilde{\Gamma }$ in the definitions (5) and (6). The positive FDR is defined by $\text{pFDR}(\tilde{\Gamma })=E\{\frac{V(\tilde{\Gamma })}{R(\tilde{\Gamma })}|R(\tilde{\Gamma })>0\}$. The following theorem is based on the generalized significance region $\tilde{\Gamma }$.

Theorem 2.1. Suppose *m* identical hypothesis tests are performed with ${\tilde{P}}_{1},\ldots ,{\tilde{P}}_{m}$ and significance region $\tilde{\Gamma }$. Let $\pi_{A}(\cdot )=1-\pi_{0}(\cdot )$. Assume that $\left(P_{1},H_{1},X_{1}),\ldots ,(P_{m},H_{m},X_{m}\right)$ are i.i.d. random vectors, where ${\tilde{P}}_{j}=P_{j}\cdot \pi_{0|\alpha }(X_{j}),\;P_{j}|H_{j},X_{j}∼(1-H_{j})\cdot F_{0}+H_{j}\cdot F_{1}$ for some null distribution $F_{0}$ and alternative distribution $F_{1}$, and $H_{j}|X_{j}∼\text{Bern}(\pi_{A}(X_{j})),\;X_{j}\overset{}{∼}F_{X}$ for j=1,…,m. Then,

Supplementary material contains the proof of Theorem 2.1.

Remark 2.1. The marginal distribution of $H_{j}$ in Theorem 2.1 is Bern($\pi_{A}$), where $\pi_{A}=1-\pi_{0}$ and $\pi_{0}=P(H_{j}=0)\;\forall j=1,\ldots ,m$. In this framework, $\left(P_{j},H_{j}\right)$ are i.i.d. random variables, where $P_{j}|H_{j}∼(1-H_{j})\cdot F_{0}+H_{j}\cdot F_{1}$ and $H_{j}∼\text{Bern}(\pi_{A})$. The standard *q*-value method is established on this modeling setup. Therefore, we can still apply the standard *q*-value method, while controlling pFDR, to the *P*-values generated from the model in Theorem 2.1.

(7) $$V_{j}(t)=1\{P_{j}\leq u_{t}(X_{j}),H_{0j}\}.$$

(8) $$\text{pFDR}(\tilde{\Gamma })=P(H_{j}=0|{\tilde{P}}_{j}\in \tilde{\Gamma })=\frac{EV(\tilde{\Gamma })}{ER(\tilde{\Gamma })},\;\forall j=1,\ldots ,m.$$

Theorem 2.1 establishes that $\text{pFDR}(t)=\frac{EV(t)}{ER(t)}$. Our estimator is obtained by estimating EV(t) and ER(t). The denominator ER(t) can be easily estimated as R(t). However, the number of false positives V(t) is unknown. To estimate the numerator EV(t), we propose to estimate EV(t) using $E\{V(t)|\vec{X}=(X_{1},\ldots ,X_{m})\}$, which is both the best predictor of V(t) under a squared error loss function and an unbiased estimator of EV(t).

When the simple null hypothesis is true, and the test statistic is continuous, the *P*-value follows a uniform distribution between 0 and 1. From this fact, we make the following assumption:

Assumption 2.1. $P_{j}|H_{j}=0∼\text{Unif}(0,1)$.

Let ${\vec{X}}_{-j}$ denote a vector $\vec{X}$ without the *j*th element. Under the model assumption described in Theorem 2.1, the following properties are obtained:

Under properties from (10) to (12) and Assumption 2.1, $E\{V(t)|\vec{X}\}$ has expression:

By combining the predetermined form of pFDR(t) and (13), the pFDR estimator is established: where $\pi_{0}(\cdot )$ and $\pi_{0|\alpha }(\cdot )$ are considered known. The pFDR estimator (15) serves as an upper bound for (14), where the equality holds when $\pi_{0|\alpha }(X_{j})\geq t$ for all *j*. We adopt the simpler version (15) as our pFDR estimator, used in the simulation study and data analysis. Then, we can define *q*-value and its estimator that can be utilized to declare genes to be DE:

Up to this point, $\pi_{0}(\cdot )$ and $\pi_{0|\alpha }(\cdot )$ have been treated as given. In practice, we must estimate both conditional null probabilities to apply our method. The following section discusses an estimating procedure.

(9) $$\begin{array}{c} (P_{j},H_{j},X_{j})⊥{\vec{X}}_{-j}\to P_{j}|\vec{X}\overset{d}{=}P_{j}|X_{j} \end{array}$$

(10) $$(P_{j},H_{j},X_{j})⊥{\vec{X}}_{-j}\to H_{j}|\vec{X}\overset{d}{=}H_{j}|X_{j}$$

(11) $$\begin{array}{c} (P_{j},H_{j},X_{j})⊥{\vec{X}}_{-j}\to P_{j}|H_{j},\vec{X}\overset{d}{=}P_{j}|H_{j},X_{j} \end{array}$$

(12) $$X_{j}⊥P_{j}|H_{j}\to P_{j}|H_{j},X_{j}\overset{d}{=}P_{j}|H_{j}.$$

(13) $$\begin{array}{c} E\{V(t)|\vec{X}\}\\ =\sum_{j=1}^{m}E\{V_{j}(t)|\vec{X}\}\;∵V(t)=\sum_{j=1}^{m}V_{j}(t)\;\;\text{and linearity}\\ =\sum_{j=1}^{m}P\{P_{j}\leq u_{t}(X_{j}),H_{0j}|\vec{X}\}\;∵(7)\\ =\sum_{j=1}^{m}P\{P_{j}\leq u_{t}(X_{j})|H_{0j},\vec{X}\}\cdot P(H_{0j}|\vec{X})\\ =\sum_{j=1}^{m}P\{P_{j}\leq u_{t}(X_{j})|H_{0j},X_{j}\}\cdot P(H_{0j}|X_{j})\;∵(10,11)\\ =\sum_{j=1}^{m}u_{t}(X_{j})\cdot \pi_{0}(X_{j})∵(12,\text{Assumption}\;2.1). \end{array}$$

(14) $$\begin{array}{c} \hat{\text{pFDR}}(t)=\frac{\sum_{j=1}^{m}u_{t}(X_{j})\cdot \pi_{0}(X_{j})}{R(t)} \end{array}$$

(15) $$\leq \frac{t}{R(t)}\cdot \sum_{j=1}^{m}\frac{\pi_{0}(X_{j})}{\pi_{0|\alpha }(X_{j})},$$

(16) $$Q_{j}=\underset{t:\;t\geq {\tilde{P}}_{j}}{\min}\;\text{pFDR}(t)\;\text{and}\;{\hat{Q}}_{j}=\underset{t:\;t\geq {\tilde{P}}_{j}}{\min}\;\hat{\text{pFDR}}(t).$$

### 2.4 Estimation of $\pi_{0}(\cdot )$ and $\pi_{0|\alpha }(\cdot )$

To simplify the problem of estimating $\pi_{0}(\cdot )$ and $\pi_{0|\alpha }(\cdot )$, we first derive a useful property. Under the model described in Theorem 2.1 and Assumption 2.1, $\pi_{0|\alpha }(\cdot )$ satisfies


(17) $$\begin{array}{cc} \pi_{0|\alpha }(X_{j})= & P(H_{0j}|P_{j}\leq \alpha ,X_{j})=\alpha \cdot \frac{\pi_{0}(X_{j})}{P(P_{j}\leq \alpha |X_{j})}. \end{array}$$

According to equality (17), when both $\pi_{0}(X_{j})$ and $P(P_{j}\leq \alpha |X_{j})$ are known, $\pi_{0|\alpha }(X_{j})$ can be obtained. Thus, we now discuss how to estimate $\pi_{0}(X_{j})$ and $P(P_{j}\leq \alpha |X_{j})$.

Let $N_{nh}$ be a user-selected neighborhood size. Let $N_{j}\subseteq \{1,\ldots ,m\}$ contain the $N_{nh}$ indices corresponding to the $N_{nh}$ genes whose covariate values are closest to $X_{j}$ in Euclidean distance. Both probabilities $\pi_{0}(X_{j})$ and $P(P_{j}\leq \alpha |X_{j})$ are estimated using only the neighborhood *P*-values $\left\{P_{i}:i\in N_{j}\right\}$. First, $\pi_{0}(X_{j})$ is estimated using the method of Nettleton *et al.* (2006) applied to $\left\{P_{i}:i\in N_{j}\right\}$, which gives where $P_{\mathrm{cut},j}$ is a threshold determined by Nettleton *et al.* (2006) such that the empirial distribution of $\left\{P_{i}:i\in N_{j},P_{i}\geq P_{\mathrm{cut},j}\right\}$ is approximately uniform. See Nettleton *et al.* (2006) for the details.


(18) $${\hat{\pi }}_{0}(X_{j})=\frac{\sum_{i\in N_{j}}1(P_{i}\geq P_{\mathrm{cut},j})}{N_{nh}}\cdot \frac{1}{1-P_{\mathrm{cut},j}},$$

Next, $P(P_{j}\leq \alpha |X_{j})$ can be easily estimated as the proportion of the *P*-values in $\left\{P_{i}:i\in N_{j}\right\}$ ≤ α:


(19) $$\hat{P}(P_{j}\leq \alpha |X_{j})=\frac{\sum_{i\in N_{j}}1(P_{i}\leq \alpha )}{N_{nh}}.$$

By (17), a natural estimator of $\pi_{0|\alpha }(X_{j})$ is ${\hat{\pi }}_{0|\alpha }(X_{j})=1\wedge \{\alpha \cdot \frac{{\hat{\pi }}_{0}(X_{j})}{\hat{P}(P_{j}\leq \alpha |X_{j})}\}$. As a result, all necessary components for our method are obtained. The following Section 2.5 provides an in-depth discussion of the rejection rule.

### 2.5 Implications of the rejection rule

To better understand our rejection rule, we derive an equivalent condition characterizing the rejection rule in terms of a conditional type 1 error posterior probability, as specified in the following theorem.

Theorem 2.2. Consider the same inference setup described in Theorem 2.1 with a rejection rule $P_{j}\leq u(X_{j})$, for a given nonnegative function u(⋅). Assume that Assumption 2.1 holds. Let $T_{1j}$ be the event that a type 1 error occurs for test *j*. If the rejection rule is more conservative than the classic rejection rule $P_{j}\leq \alpha$, i.e. $\underset{j}{\max}u(X_{j})\leq \alpha$, then,

The proof is included in the supplementary material. According to Theorem 2.2, among more conservative rejection rules than the classic rejection rule, the rejection rule that preserves constant type 1 error posterior probability given the low *P*-value condition and covariate variables $\vec{X}$ is uniquely determined by $u(X_{j})=\frac{t}{\pi_{0|\alpha }(X_{j})}$ for some *t*. In other words, under the conservativeness condition, our proposed rejection rule is the only one that equalizes the conditional type 1 error posterior probability across all tests. According to the model assumed in Theorem 2.2, rejection situations vary by covariate variables. A rejection rule ignoring the distinct situations is incapable of equalizing error control as described in Theorem 2.2. However, our rejection rule ensures constant conditional type 1 error posterior probabilities across all tests, contrary to traditional rejection rules.

$$\begin{array}{c} P(T_{1j}|P_{j}\leq \alpha ,\vec{X})\;\text{is the same for all}\;j=1,\ldots ,m\\ \Leftrightarrow u(X_{j})=\frac{t}{\pi_{0|\alpha }(X_{j})}\;\text{for all}\;j=1,\ldots ,m\;\text{and some}\;t>0. \end{array}$$

## 3 Simulation study

### 3.1 Model description

We conduct a simulation study to assess our method’s performance, inspired by the model in Theorem 2.1. We consider gene expression datasets with *m*=10 000 genes generated independently from normal distributions with gene-specific variance from an inverse chi-square distribution. The covariate variable, which affects the probability of being an EE gene, is denoted by *X* and assumed to be normally distributed. A DE gene’s treatment effect is randomly generated from a normal distribution. Let *j* and *k* be the gene and treatment group indices, respectively. Let *s* denote a sample index within a treatment group. The sample size within a treatment group *n* is set to 10. Then, the data model with $Y_{sk}^{j}$ as the response variable is described as follows: and independence among all random variables holds except where indicated otherwise by conditioning. After generating the dataset from (20), a two-sample *t*-test is used to obtain a *P*-value for testing each gene’s treatment effect.


(20) $$\begin{array}{c} Y_{sk}^{j}\;|\;\delta_{k}^{j},\sigma_{j}^{2}∼N(\delta_{k}^{j},\;\sigma_{j}^{2}),\;\text{where}\;j\in \{1,\ldots m\}\;\text{and}\;s\in \{1,\ldots n\}\\ \delta_{0}^{j}=0\;\text{and}\;\delta_{1}^{j}|H_{j}=(1-H_{j})\cdot 0+H_{j}\cdot N(\mu_{\delta },\;\sigma_{\delta }^{2}=0.02^{2})\\ H_{j}|X_{j}∼\text{Bern}(\pi_{A}(X_{j})),\text{where}\;\pi_{A}(X_{j})=1-\pi_{0}(X_{j}),\\ X_{j}∼N(\mu_{X}=4,\sigma_{X}^{2}=0.5^{2}),\sigma_{j}^{2}∼\text{Inv}-\chi_{5}^{2}, \end{array}$$

**Figure 2.**
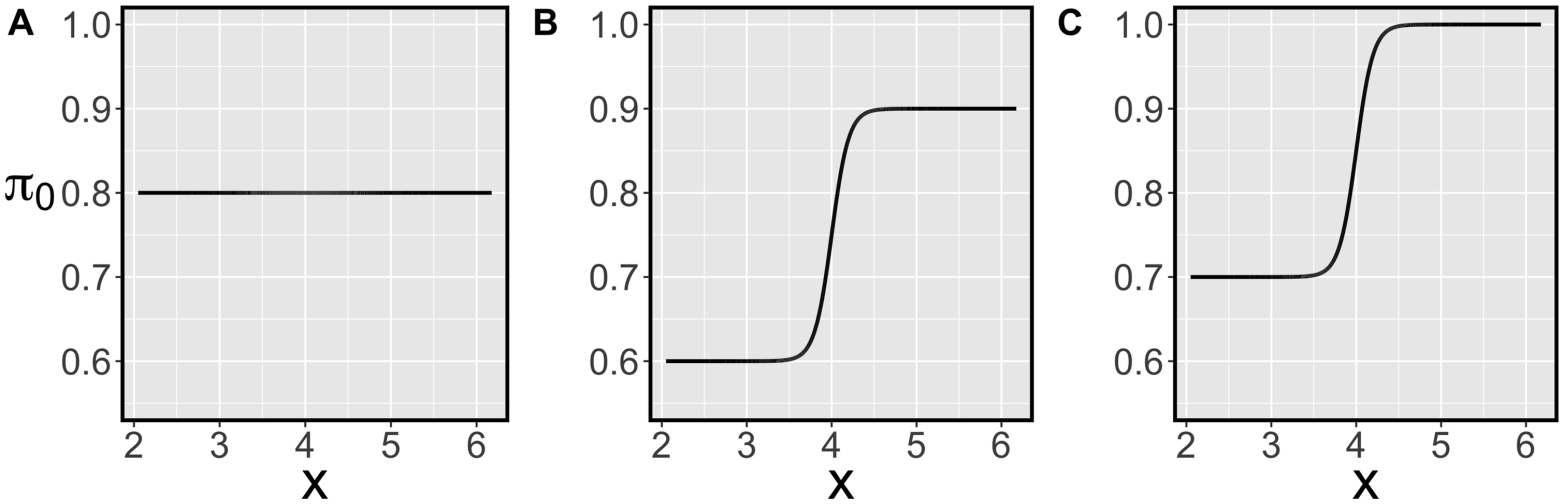
Functions from (A) to (C) illustrate three $\pi_{0}(x)$ functions used in the simulation, where $\pi_{0}^{A}(x)=0.8$, $\pi_{0}^{B}(x)=0.6+\frac{0.3}{1+\text{exp}\;\{-10\cdot (x-4)\}}$, and $\pi_{0}^{C}(x)=0.7+\frac{0.3}{1+\text{exp}\;\{-10\cdot (x-4)\}}$.

The simulation is conducted with different combinations of $\mu_{\delta }$ and $\pi_{0}(\cdot )$. $\mu_{\delta }$ is chosen from a set of four equally spaced values from 0.15 to 0.24. Three $\pi_{0}(\cdot )$ functions are considered, illustrated in Fig. 2. The function $\pi_{0}^{A}(\cdot )$ is a constant function, whereas $\pi_{0}^{B}(\cdot )$ and $\pi_{0}^{C}(\cdot )$ are increasing sigmoid functions. Using $\pi_{0}^{A}(\cdot )$, we determine whether the proposed method works well when the probability of being an EE gene does not vary with the gene-specific covariate. Using $\pi_{0}^{B}(\cdot )$ and $\pi_{0}^{C}(\cdot )$, we determine whether the proposed method performs better than other methods when the true model follows the working model. $\pi_{0}^{C}(\cdot )$ has a more extreme characteristic than $\pi_{0}^{B}(\cdot )$ due to a covariate region with a null probability of one.

### 3.2 Methods description

Under a target FDR level of 0.05, the proposed method is compared to the standard *q*-value, IHW, BL, and AdaPT methods. These methods are chosen because they enable precise control of the FDR in the simulation study of Korthauer *et al.* (2019).

Let us begin by discussing the tuning parameters of the proposed method: $N_{nh}$ and α. $N_{nh}$ is set to 2000. The value of α is chosen arbitrarily or through cross-validation (cv). First, we choose α values of 0.05 and 1 to better understand the proposed method’s properties. In addition, when α equals 1, we include the proposed method with true null probability $\pi_{0}(\cdot )$ for a reference. Depending on whether the true $\pi_{0}(\cdot )$ is used (true) or whether $\pi_{0}(\cdot )$ is estimated (est), and on the value of α, the proposed method’s procedures are referred to as prop.q(true, α = 1), prop.q(est, α = 1), prop.q(est, α = 0.05), and prop.q(est, α = cv).

The latter approach is our suggested α selection procedure based on repeated 10-fold cross-validation that maximizes the expected number of DDE genes, described as follows. We partition the observations $\left\{(X_{j},P_{j}):j=1,\ldots ,m\right\}$ completely at random into 10 parts. Holding each part out as a test set in turn, the other nine parts are used as a training set. For each of 100 equally spaced α values between 0.001 and 0.2, the training data are used to estimate $\pi_{0|\alpha }(\cdot )$ and our rejection rule for controlling pFDR at the target level 0.05. The number of DDE genes is determined based on applying the estimated rejection rule to the test data. This entire 10-fold cross-validation process is repeated M times, and the average number of DDE genes across the 10 ×*M* test sets is determined for each value of α. The value of α with the highest average number of DDE genes is selected and used with our proposed procedure on the entire dataset to identify differentially expressed genes. In the simulation study, we use *M* = 1, while *M* = 100 in the data analysis section.

As discussed in Remark 2.1, the standard *q*-value method is still applicable in our simulation setup and is guaranteed to control pFDR. To estimate $\pi_{0}=P(H=0)$, the histogram-based method (Nettleton *et al.* 2006) is used. Moreover, $\pi_{0}$ can be easily approximated by $P(H_{1}=0)=E_{X_{1}}P(H_{1}=0|X_{1})\approx \frac{\sum_{j=1}^{m}P(H_{j}=0|X_{j})}{m}=\frac{\sum_{j=1}^{m}\pi_{0}(X_{j})}{m}$. Depending on whether the true parameter is used or not, the standard *q*-value method’s procedures are referred to as std.q(true) and std.q(est). For simplicity, the omission of the estimator and true parameter symbols indicates the estimator version of the procedure with parameters estimated from data. For example, std.q = std.q(est).

Lastly, we turn to the IHW, BL, and AdaPT methods implemented in R packages IHW, swfdr, and adaptMT. IHW and swfdr are Bioconductor R packages, and adaptMT is a CRAN R package. Essentially, we follow the default configuration of the packages. For the AdaPT method, inspired by the simulation results in Korthauer *et al.* (2019), we use the adapt_glm function with the settings specified in the article. The procedures associated with the three methods are denoted by their respective names. In total, nine procedures are compared. The simulation results are analyzed without the procedures that use true parameter values because these methods cannot be used in practice.

### 3.3 Simulation results

The nine procedures are compared in terms of mean false discovery proportion, mean true positive number, mean area under the receiver-operating characteristic (ROC) curve (AUC), and mean partial area under the ROC curve (pAUC). The ROC curve displays the trade-off between true-positive rate and false-positive rate. AUC and pAUC are the ROC curve’s summary statistics, calculated based on each procedure’s adjusted *P*-values or *q*-values. High AUC and pAUC values indicate that the procedure generally prioritizes true positives over false positives. The pAUC value is calculated by the standardized area under the ROC curve with a false-positive rate ≤ 0.1, regarded as a relevant region in our inference situation.

For each scenario composed of $\mu_{\delta }$ and $\pi_{0}(\cdot )$, we generated 5000 datasets, which were used to approximate the four mean values: mean false discovery proportion, mean true positive number, mean AUC, and mean pAUC, denoted by $\bar{V/R}$, $\bar{S}$, $\bar{\text{AUC}}$, and $\bar{\text{pAUC}}$. When a procedure declares no significant hypotheses, the false discovery proportion is set to zero, which means $\bar{V/R}$ is an empirical estimate of FDR rather than pFDR. However, in all our simulation scenarios, the probabilities of discovery corresponding to our proposed procedures are ∼1. Therefore, for our proposed procedures, FDR ≈ pFDR in our simulation.

**Figure 3.**
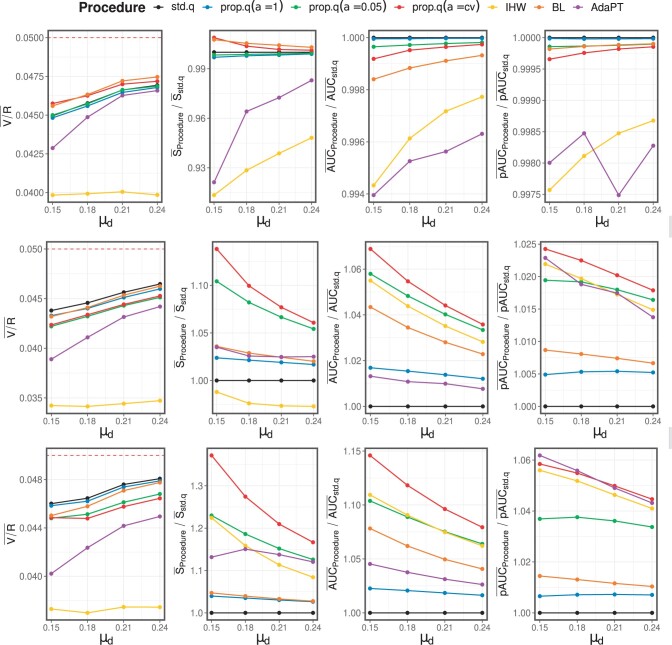
Each row contains four graphs depicting the summary statistics of $\bar{V/R}$, $\bar{S}$, $\bar{\text{AUC}}$, and $\bar{\text{pAUC}}$, derived from the scenarios of $\pi_{0}^{A}(\cdot )$ (top), $\pi_{0}^{B}(\cdot )$ (middle), and $\pi_{0}^{C}(\cdot )$ (bottom), respectively.

Supplementary Table S1A–C summarizes all the simulation results. Figure 3 illustrates the results associated with the functions $\pi_{0}^{A}(\cdot )$, $\pi_{0}^{B}(\cdot )$, and $\pi_{0}^{C}(\cdot )$, respectively. In the figures, except for $\bar{V/R}$, the ratio to std.q is calculated to illustrate the relative performance. Above all, all procedures under consideration control FDR in all scenarios.

Let us discuss the $\pi_{0}^{A}(\cdot )$ results. As illustrated in Fig. 3, all procedures have nearly identical $\bar{\text{AUC}}$ and $\bar{\text{pAUC}}$ across all scenarios, showing that they perform similarly in terms of prioritizing true discoveries. In terms of true positive number $\bar{S}$, when $\mu_{\delta }$ is small, the std.q outperforms the IHW and AdaPT. On the other hand, all procedures associated with the proposed method perform nearly identically to the std.q, which is understandable as the proposed method generalizes the standard *q*-value method. The results of $\pi_{0}^{A}(\cdot )$ suggest that the proposed method performs as well as std.q even when the covariate is irrelevant.

We now turn to the $\pi_{0}^{B}(\cdot )$ and $\pi_{0}^{C}(\cdot )$ results. First, we explore the proposed method’s properties by comparing the related procedures to std.q. The summary statistics $\bar{S}$, $\bar{\text{AUC}}$, and $\bar{\text{pAUC}}$ all indicate the same conclusion. The procedure performs best in the order of prop.q(α=cv), prop.q(α=0.05), prop.q(α=1), then std.q. The order is well illustrated in Fig. 3. Since prop.q(α=1) is better than std.q, we can conclude that there is an improvement by considering covariate-specific null probability. It is noteworthy that the BL method consistently outperforms prop.q(α=1), even though both methods use the covariate-specific null probability. By comparing prop.q(α=0.05) and prop.q(α=1), we can conclude that incorporating the classic rejection rule improves the proposed method. From the comparison between prop.q(α=cv) and prop.q(α=0.05), it can be concluded that cross-validation is beneficial for α selection to improve all evaluation criteria. As a result, we recommend using cross-validation to determine the value of α and setting the default value to 0.05.

The prop.q(α=cv) method is now compared to IHW, BL, and AdaPT. In terms of $\bar{S}$ and $\bar{\text{AUC}}$, prop.q(α=cv) surpasses other procedures in all scenarios. In the case of the AdaPT method, we can see that the performance is weakened in terms of $\bar{\text{AUC}}$, which is likely due to the vulnerability stated in Section 1. As seen in Fig. 3, there are scenarios where AdaPT method outperforms prop.q(α=cv) regarding $\bar{\text{pAUC}}$. For the corresponding scenarios, however, prop.q(α=cv) consistently generates more true positives $\bar{S}$ than AdaPT, which may be attributed to the differing FDR estimators. Based on our simulation setup, we can conclude that the proposed method using cross-validation to select α outperforms the competing FDR-controlling methods in most scenarios and evaluation criteria that we considered.

The supplementary material depicts additional simulations under various conditions, including scenarios with a multi-modal null probability function, covariates generated from a mixed normal distribution, and correlated *P*-values. Supplementary Figure S4 demonstrates the validity of our method to maintaining FDR control and power under a distinct shape of null probability function and the covariate distribution. In addition, we investigated a simulation in which gene expressions are correlated. As shown in Supplementary Fig. S5, when the correlation is relatively small, there are no problems with FDR control or true discovery capability. As with other methods, we observed that FDR levels become higher than the nominal rate when the correlation is relatively high.

## 4 Data analysis

We tested our proposed method using RNA-seq data regarding disease resilience in young, healthy pigs (Lim *et al.* 2021), and additional data on gene lengths. A comprehensive description of the study’s design and hypotheses testing is described in Lim *et al.* (2021), which is summarized as follows. The study enrolled 912 F1 barrows at ∼27 days of age in 15 batches. After three weeks in a healthy quarantine nursery, the piglets were exposed to natural polymicrobial diseases found on commercial farms. Not only were gene expression levels of the piglets’ blood samples quantified, but also disease resilience phenotypes such as subjective health score, treatment rate, mortality, and growth rate. Although the article (Lim *et al.* 2021) tested numerous hypotheses, our current article focuses on the association between gene expression and concurrent growth rate using blood samples taken during quarantine nursery periods before disease exposure. We anticipated that the disease-independent growth rate would be a long-term physical process, which is expected to be associated with the expression of longer genes. This expectation motivated us to concentrate on the association involving growth rate before disease exposure.

**Figure 4.**
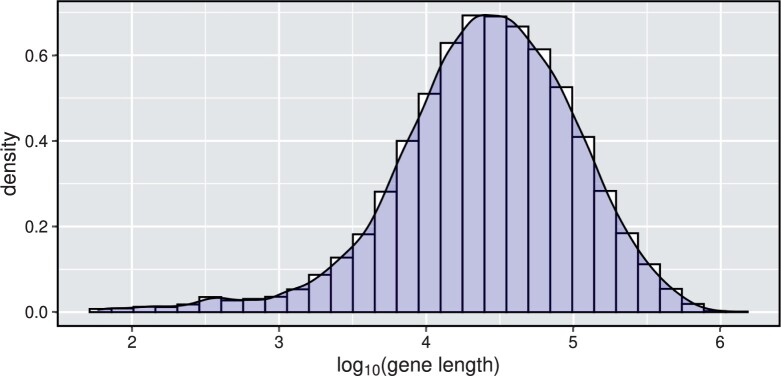
The histogram of log10 transformed gene length for 10 858 genes. The log10 transformed gene lengths have a mean of 4.4 and a standard deviation of 0.61.

The following is the analysis we conducted. The gene expression in blood samples acquired during quarantine nursery was quantified using 3ʹmRNA sequencing with a globin block. Using the data in Lim *et al.* (2021) and genes in the Ensembl database, we analyzed 10 858 genes with a nonzero read count for at least 80% of the samples. The growth rate of a pig was used as a common dependent variable. We used log-scale read counts normalized and adjusted for nuisance factors as described by Lim *et al.* (2021). A *P*-value was calculated for each gene, testing whether the adjusted log2 transformed read count has a zero slope coefficient. In total, we generated 10 858 *P*-values. Figure 4 illustrates the histogram of log10-transformed gene lengths utilized to determine the covariate distribution in the simulation discussed in Section 3.

We applied the seven procedures, described in Section 3, to the *P*-values and their associated gene lengths. The number of significant tests at various nominal FDR levels are summarized in Table 1. Regarding the proposed method, decreasing α from 1 to 0.05 or using cross-validation to select α tended to increase the number of significant tests, consistent with the simulation outcome. Furthermore, prop.q(α=cv) consistently declared a greater or similar number of tests significant than the std.q, IHW, and BL methods. Except for the nominal level of 0.1, the prop.q(α=cv) generated more significant results than AdaPT. When the nominal level is set to 0.01, the AdaPT method declared no tests significant.

**Table 1.** Summary of the number of tests declared to be significant by the seven procedures at four nominal FDR levels 0.01, 0.05, 0.1, and 0.2.

Level	Std.q	Prop.q (α=1)	Prop.q (α=0.05)	Prop.q (α=cv)	IHW	BL	AdaPT
0.01	181	182	182	184	185	184	0
0.05	298	298	305	306	290	299	291
0.10	419	425	442	443	385	426	455
0.20	707	753	774	774	608	736	725

**Figure 5.**
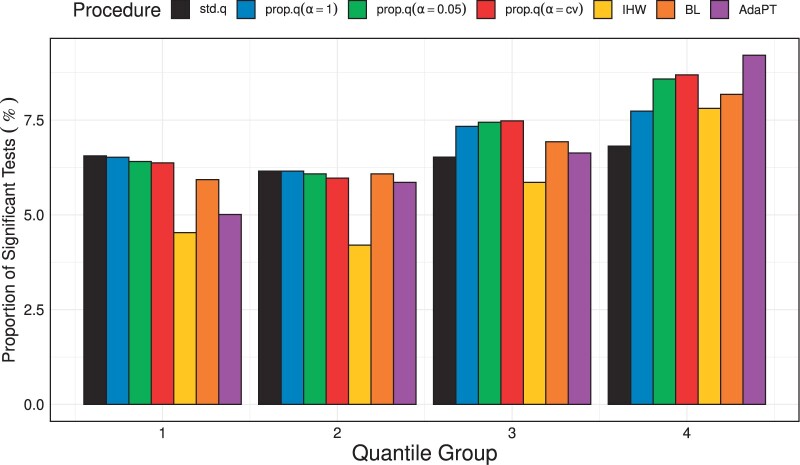
Barplot depiction of the proportion of tests declared to be significant by the three procedures at a nominal pFDR level of 0.2 for four gene length-based groups with almost equal numbers.

**Figure 6.**
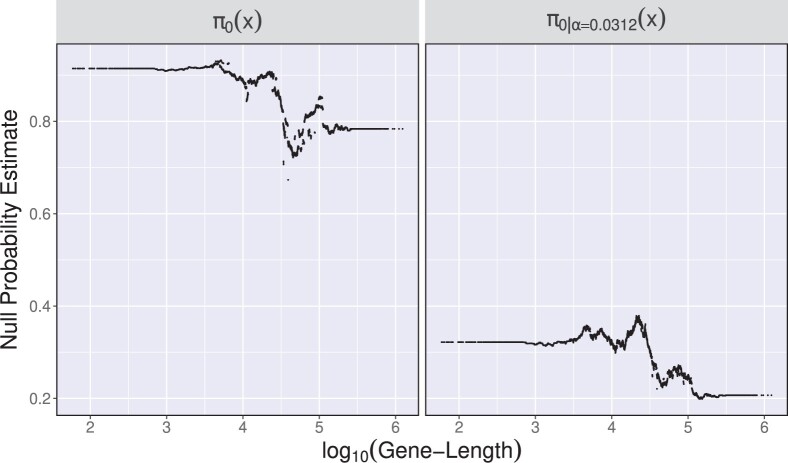
Null probability estimates of $\pi_{0}(x)$ and $\pi_{0|\alpha }(x)$ for 10 858 covariate values, following the procedure explained in Section 2.4. The nominal pFDR level is set to 0.2. An α value of 0.0312 was chosen through the cross-validation approach.

To observe additional patterns genes are classified into four groups according to their lengths. As illustrated in Fig. 5, regardless of procedures, the significantly declared tests are observed in greater abundance in the 4th quantile group than in all other quantile groups. The number of significant tests increases gradually from the second quantile group. The finding supports our intuition that the growth rate is a long-term physical process that tends to involve longer genes and supports our method’s assumption that the null probability varies with gene length. The null probability estimates described in Fig. 6 also shows a tendency supporting the assumption.

We conducted gene set enrichment analyses (GSEA) with preranked, adjusted *P*-values to bring biological significance to the data analysis. Conducting the GSEA using the adjusted *P*-value, not the raw *P*-value, makes sense as gene length provides additional information regarding the genes of interest. Using the same FDR threshold of 0.05 for the enriched terms, our method declares a comparable number or more biological processes significant compared to std.q, BL, and IHW (Supplementary Fig. S1A). In addition, Supplementary Fig. S1B demonstrates that the *P*-values derived from GSEA with prop.q(α = cv) are generally lower than the *P*-values derived from GSEA with other methods, indicating that our method may have a greater power to discover meaningful biological processes. The simulation result indicates that our method consistently outperforms other approaches regarding AUC and that gene set enrichment testing with preranked GSEA can be advantageous, which supports the result illustrated in Supplementary Fig. S1B. The application of AdaPT to the data reveals that AdaPT generates a large number of duplicate adjusted *P*-values, thereby limiting the enrichment test; therefore, we excluded the GSEA results with the AdaPT method.

Additional data analysis is performed using four gene expression datasets described in Lei and Fithian (2018) and available at https://github.com/lihualei71/adaptPaper/tree/master/data. Each dataset is named after the corresponding file name. Supplementary Table S2 presents the results, showing that, except for AdaPT, our approach consistently declares more tests significant than other methods. Moreover, there is a substantial overlap between our approach and the AdaPT method in terms of the significantly declared tests. The null probability functions estimated from these data analyses (depicted in Supplementary Fig. S3) show that a sigmoidal shape may be a common form for the null probability function.

## 5 Discussion

While the proposed method demonstrates significant gains over existing methods, there are still areas for improvement. First, the modeling framework upon which our method is developed is generalizable. One may consider a method in which the alternative distribution $F_{1}$ varies with the covariate variable. Second, the estimation procedure for estimating the null probabilities can be improved. The simulation results indicate that the BL method consistently beats our method with α=1, indicating a promising direction for further development of the estimation procedure. Finally, different rejection rules can be defined using different posterior probability types. Performance is predicted to vary according to the target posterior probability. We anticipate that subsequent studies will examine our method from various perspectives.

The data analysis demonstrates that the estimation of null probabilities provides valuable insights into the relationship between predictors and the features of interest. These estimates are meaningful on their own and can be utilized effectively. For instance, clustering features based on estimates can reveal additional research areas of interest. Moreover, conditional null probability estimates can specify genes relevant to specific biological processes, essential for gene-set enrichment analysis.

## Supplementary Material

btad498_Supplementary_Data

## Data Availability

The data were generated on commercially owned animals and, therefore, contain proprietary information. They can be made available by the corresponding author upon reasonable request. The data analyzed in the supplemental materials can be found at https://github.com/hsjeon1217/conditional_method.
