# Supplementary material for: Adjusting for gene-specific covariates to improve RNA-seq analysis
Source: Bioinformatics. 2023 Aug 17;39(8):btad498. doi: 10.1093/bioinformatics/btad498 (PMC10460482; doi:10.1093/bioinformatics/btad498)
Supplement: btad498_Supplementary_Data [file btad498_supplementary_data.pdf]

Supplementary Material

2023-06-12

1 Proof of Theoretical Results

1.1 Proof of Theorem 2.1

Proof. The equality is derived as follows:

$$
\begin{aligned}
\mathbb{E}\Big[\frac{V(\tilde{\Gamma})}{R(\tilde{\Gamma})}\Big|R(\tilde{\Gamma})>0\Big] &= \sum_{k=1}^{m}\mathbb{E}\Big[\frac{V(\tilde{\Gamma})}{R(\tilde{\Gamma})}\Big|_{R(\tilde{\Gamma})=k,\, R(\tilde{\Gamma})>0}\Big]\cdot\mathbb{P}(R(\tilde{\Gamma})=k\mid R(\tilde{\Gamma})>0)\\
&= \sum_{k=1}^{m}\mathbb{E}\Big[\frac{V(\tilde{\Gamma})}{k}\Big|R(\tilde{\Gamma})=k\Big]\cdot\mathbb{P}(R(\tilde{\Gamma})=k\mid R(\tilde{\Gamma})>0)\\
&\overset{(\star)}{=} \mathbb{P}(H_j=0\mid \tilde{P}_j\in\tilde{\Gamma})\cdot\mathbb{P}(R(\tilde{\Gamma})>0\mid R(\tilde{\Gamma})>0)\\
&= \mathbb{P}(H_j=0\mid \tilde{P}_j\in\tilde{\Gamma})\\
&= \frac{m\cdot\mathbb{P}(H_j=0,\tilde{P}_j\in\tilde{\Gamma})}{m\cdot\mathbb{P}(\tilde{P}_j\in\tilde{\Gamma})}\\
&= \frac{\mathbb{E}V(\tilde{\Gamma})}{\mathbb{E}R(\tilde{\Gamma})},
\end{aligned}
$$

where $(\star)$ follows because

$$
\begin{aligned}
\mathbb{E}[V(\tilde{\Gamma})\mid R(\tilde{\Gamma})=k] &= \mathbb{E}\Big[\sum_{i=1}^{m}1(H_i=0)1(\tilde{P}_i\in\tilde{\Gamma})\mid {}_{\tilde{P}_1,\ldots,\tilde{P}_k\in\tilde{\Gamma},\,\tilde{P}_{k+1},\ldots,\tilde{P}_m\notin\tilde{\Gamma}}\Big]\\
&= \mathbb{E}\Big[\sum_{i=1}^{k}1(H_i=0)\mid {}_{\tilde{P}_1,\ldots\tilde{P}_k\in\tilde{\Gamma},\,\tilde{P}_{k+1},\ldots\tilde{P}_m\notin\tilde{\Gamma}}\Big]\\
&= \sum_{i=1}^{k}\mathbb{P}\Big[H_i=0\mid {}_{\tilde{P}_1,\ldots\tilde{P}_k\in\tilde{\Gamma},\,\tilde{P}_{k+1},\ldots\tilde{P}_m\notin\tilde{\Gamma}}\Big]\\
&= k\cdot\mathbb{P}(H_j=0\mid\tilde{P}_j\in\tilde{\Gamma})\ \because \text{i.i.d.}
\end{aligned}
$$

□

1.2 Proof of Theorem 2.2

Proof. Let H_{0j} denote $H_j = 0$.

$$\begin{aligned}
\mathbb{P}(\,T_{1j} \mid P_j \le \alpha, \vec{X}) &= \mathbb{P}\big(P_j \le u(X_j), H_{0j} \mid P_j \le \alpha, \vec{X}\big) \quad \because (7) \\
&= \mathbb{P}\big(P_j \le u(X_j) \mid H_{0j}, P_j \le \alpha, \vec{X}\big) \cdot \mathbb{P}\big(H_{0j} \mid P_j \le \alpha, \vec{X}\big) \\
&= \frac{\mathbb{P}\big(P_j \le u(X_j), P_j \le \alpha \mid H_{0j}, \vec{X}\big)}{\mathbb{P}\big(P_j \le \alpha \mid H_{0j}, \vec{X}\big)} \cdot \frac{\mathbb{P}\big(H_{0j}, P_j \le \alpha \mid \vec{X}\big)}{\mathbb{P}\big(P_j \le \alpha \mid \vec{X}\big)} \\
&= \frac{\mathbb{P}\big(P_j \le u(X_j) \wedge \alpha \mid H_{0j}, \vec{X}\big)}{\mathbb{P}\big(P_j \le \alpha \mid H_{0j}, \vec{X}\big)} \cdot \frac{\mathbb{P}\big(P_j \le \alpha \mid H_{0j}, \vec{X}\big) \cdot \mathbb{P}\big(H_{0j} \mid \vec{X}\big)}{\mathbb{P}\big(P_j \le \alpha \mid \vec{X}\big)} \\
&= \frac{\mathbb{P}\big(P_j \le u(X_j) \wedge \alpha \mid H_{0j}, \vec{X}\big)}{\mathbb{P}\big(P_j \le \alpha \mid H_{0j}, \vec{X}\big)} \cdot \frac{\mathbb{P}\big(P_j \le \alpha \mid H_{0j}, X_j\big) \cdot \mathbb{P}\big(H_{0j} \mid X_j\big)}{\mathbb{P}\big(P_j \le \alpha \mid X_j\big)} \quad \because (9, 10, 11) \\
&= \frac{u(X_j) \wedge \alpha}{\alpha} \cdot \mathbb{P}\big(H_{0j} \mid P_j \le \alpha, X_j\big) \quad \because (11, 12, \text{Assumption } 2.1) \\
&= \frac{u(X_j)}{\alpha} \cdot \pi_{0|\alpha}(X_j) \quad \because \max_j u(X_j) \le \alpha \ -(\star\star)
\end{aligned}$$

From the equality $(\star\star)$, the property that $\mathbb{P}(\,T_{1j} \mid P_j \le \alpha, \vec{X})$ is constant is equivalent to $u(X_j) \propto \frac{1}{\pi_{0|\alpha}(X_j)}$, with respect to j.

□

Table S1.A: Summary of statistics of $\overline{\text{V/R}}$, $\overline{\text{S}}$, $\overline{\text{AUC}}$, and $\overline{\text{pAUC}}$ derived for $\pi_0^A(\cdot)$ and different values of μ_δ.

μ_δ	Label	std.q(true)	std.q	prop.q(true,α=1)	prop.q(α=1)	prop.q(α=0.05)	prop.q(α=cv)	IHW	BL	AdaPT
0.15	$\overline{V/R}$	0.050	0.045	0.050	0.045	0.045	0.046	0.040	0.046	0.043
·	$\overline{S}$	201	188	201	187	188	190	172	189	173
·	$\overline{AUC}$	0.744	0.744	0.744	0.744	0.744	0.743	0.740	0.743	0.740
·	$\overline{pAUC}$	0.657	0.657	0.657	0.657	0.656	0.656	0.655	0.656	0.655
0.18	$\overline{V/R}$	0.050	0.046	0.050	0.046	0.046	0.046	0.040	0.046	0.045
·	$\overline{S}$	370	355	370	355	355	357	330	357	343
·	$\overline{AUC}$	0.785	0.785	0.785	0.785	0.785	0.784	0.782	0.784	0.781
·	$\overline{pAUC}$	0.697	0.697	0.697	0.697	0.697	0.697	0.696	0.697	0.696
0.21	$\overline{V/R}$	0.050	0.047	0.050	0.046	0.047	0.047	0.040	0.047	0.046
·	$\overline{S}$	547	533	547	532	533	534	500	535	518
·	$\overline{AUC}$	0.818	0.818	0.818	0.818	0.818	0.818	0.816	0.817	0.815
·	$\overline{pAUC}$	0.734	0.734	0.734	0.734	0.734	0.734	0.733	0.734	0.732
0.24	$\overline{V/R}$	0.050	0.047	0.050	0.047	0.047	0.047	0.040	0.047	0.047
·	$\overline{S}$	715	703	715	702	703	704	667	705	691
·	$\overline{AUC}$	0.845	0.845	0.845	0.845	0.845	0.845	0.843	0.845	0.842
·	$\overline{pAUC}$	0.766	0.766	0.766	0.766	0.766	0.766	0.765	0.766	0.765

Table S1.B: Summary of statistics of $\overline{\text{V/R}}$, $\overline{\text{S}}$, $\overline{\text{AUC}}$, and $\overline{\text{pAUC}}$ derived for $\pi_0^B(\cdot)$ and different values of μ_δ.

μ_δ	Label	std.q(true)	std.q	prop.q(true,α=1)	prop.q(α=1)	prop.q(α=0.05)	prop.q(α=cv)	IHW	BL	AdaPT
0.15	$\overline{V/R}$	0.050	0.044	0.050	0.043	0.042	0.042	0.034	0.043	0.039
·	$\overline{S}$	297	274	316	280	302	311	270	283	283
·	$\overline{AUC}$	0.744	0.744	0.768	0.756	0.787	0.795	0.785	0.776	0.754
·	$\overline{pAUC}$	0.657	0.657	0.663	0.660	0.669	0.672	0.671	0.662	0.672
0.18	$\overline{V/R}$	0.050	0.045	0.050	0.044	0.043	0.043	0.034	0.044	0.041
·	$\overline{S}$	526	500	550	510	541	549	488	514	513
·	$\overline{AUC}$	0.785	0.785	0.805	0.797	0.823	0.828	0.819	0.812	0.793
·	$\overline{pAUC}$	0.697	0.697	0.704	0.701	0.711	0.713	0.711	0.703	0.710
0.21	$\overline{V/R}$	0.050	0.046	0.050	0.045	0.044	0.044	0.034	0.045	0.043
·	$\overline{S}$	755	731	782	745	780	787	711	749	749
·	$\overline{AUC}$	0.818	0.818	0.835	0.830	0.851	0.854	0.847	0.841	0.826
·	$\overline{pAUC}$	0.734	0.734	0.740	0.738	0.747	0.749	0.746	0.739	0.747
0.24	$\overline{V/R}$	0.050	0.046	0.050	0.046	0.045	0.045	0.035	0.046	0.044
·	$\overline{S}$	968	947	995	963	999	1005	921	966	971
·	$\overline{AUC}$	0.845	0.845	0.860	0.856	0.874	0.876	0.869	0.865	0.852
·	$\overline{pAUC}$	0.766	0.766	0.772	0.770	0.779	0.780	0.777	0.771	0.776

Table S1.C: Summary of statistics of $\overline{\text{V/R}}$, $\overline{\text{S}}$, $\overline{\text{AUC}}$, and $\overline{\text{pAUC}}$ derived for $\pi_0^C(\cdot)$ and different values of μ_δ.

μ_δ	Label	std.q(true)	std.q	prop.q(true,α=1)	prop.q(α=1)	prop.q(α=0.05)	prop.q(α=cv)	IHW	BL	AdaPT
0.15	$\overline{V/R}$	0.050	0.046	0.050	0.046	0.045	0.045	0.037	0.045	0.040
·	$\overline{S}$	122	116	132	121	143	159	142	121	131
·	$\overline{AUC}$	0.744	0.744	0.776	0.761	0.821	0.852	0.825	0.802	0.778
·	$\overline{pAUC}$	0.656	0.656	0.665	0.661	0.681	0.695	0.693	0.666	0.697
0.18	$\overline{V/R}$	0.050	0.046	0.049	0.046	0.045	0.045	0.037	0.046	0.042
·	$\overline{S}$	236	229	252	237	272	292	266	238	264
·	$\overline{AUC}$	0.785	0.785	0.812	0.801	0.854	0.878	0.856	0.833	0.814
·	$\overline{pAUC}$	0.697	0.697	0.706	0.702	0.723	0.735	0.733	0.706	0.736
0.21	$\overline{V/R}$	0.050	0.048	0.050	0.047	0.046	0.046	0.037	0.047	0.044
·	$\overline{S}$	363	356	381	367	410	431	397	368	405
·	$\overline{AUC}$	0.818	0.818	0.841	0.833	0.880	0.897	0.880	0.859	0.844
·	$\overline{pAUC}$	0.734	0.734	0.742	0.739	0.760	0.771	0.768	0.742	0.770
0.24	$\overline{V/R}$	0.050	0.048	0.050	0.048	0.047	0.046	0.037	0.048	0.045
·	$\overline{S}$	486	480	506	493	541	560	521	493	538
·	$\overline{AUC}$	0.845	0.845	0.865	0.859	0.899	0.913	0.898	0.880	0.868
·	$\overline{pAUC}$	0.766	0.766	0.774	0.771	0.792	0.800	0.797	0.774	0.799

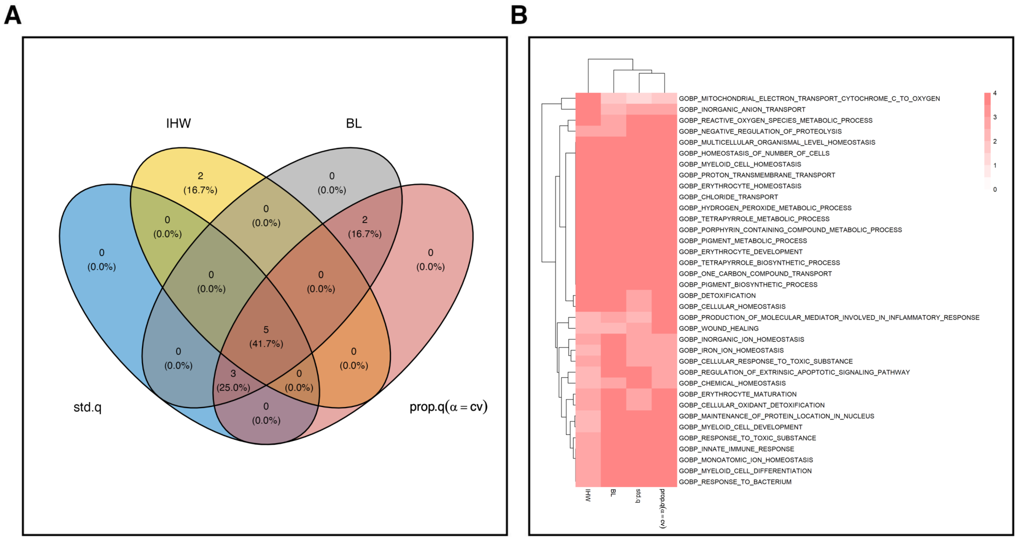

Figure S1: Figure A depicts a Venn diagram of significant biological processes (BPs) identified by the methods using an FDR threshold of 0.05. Figure B depicts a heatmap of -log p values for Gene Ontology BPs whose p-value was observed at least once to be less than 0.001 when executing gene set enrichment analysis with pre-ranked genes based on the four selected methods.

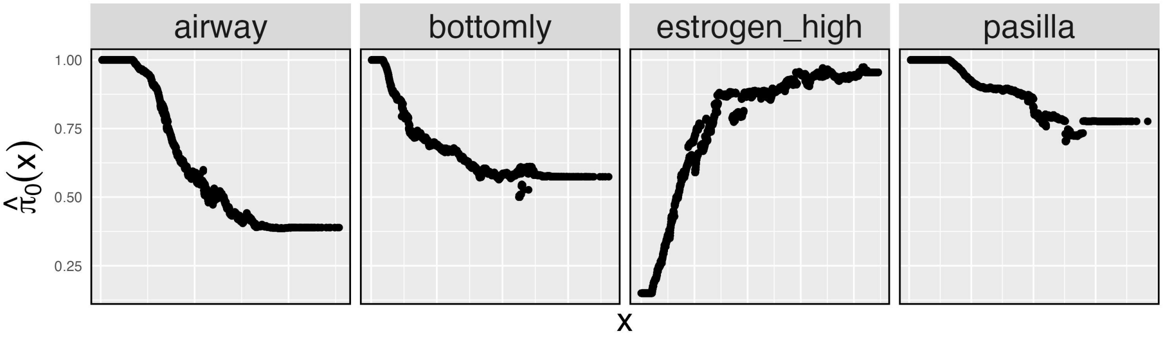

Figure S2: Four graphs illustrating estimates of the null probability of $\pi_0(x)$ obtained by applying our method to additional data sets.

Table S2: The number of tests declared significant by the seven procedures at a nominal FDR level of 0.2 when applied to four additional data sets.

Data	std.q	prop.q(α=1)	prop.q(α=0.05)	prop.q(α=cv)	IHW	BL	AdaPT
airway	5182	6000	7316	7629	6069	6557	7684
bottomly	2505	2807	3199	3255	2480	3799	3297
estrogen_high	2	1034	2237	2560	631	506	2721
pasilla	944	994	1096	1173	1056	1016	1185

2 Additional Simulation Study

Our proposed method controls pFDR as validated by Theorem 2.1, which holds for any distributional form of X as well as any functional form of $\pi_0(X)$. A key condition for Theorem 2.1 is Assumption 2.1 which states that each p-value follows uniform(0,1) distribution under the null hypothesis. It is straightforward to see from the formula (13) in the main text that our method is still expected to control pFDR well if a weaker assumption than Assumption 2.1 holds: the p-value is super-uniform, or equivalently, stochastically larger than the uniform(0, 1) under the null hypothesis. The proposed method is expected to be more conservative and have low power to detect differentially expressed genes when the level of departure from uniform(0,1) increases, as demonstrated in Table S3.

Table S3: The table demonstrates the impact on our methods when there is a divergence from the assumption that F_0 is uniform. Based on the model assumption in Theorem 2.1, we explore two simulation setups where F_0 and F_1 follow distinct Beta distributions. F_1 always follows a Beta(1, 50) distribution, while F_0 follows a Beta(1, 2) distribution in cases representing a super uniform distribution and Unif(0,1) in others. The methods are assessed using the main text's criteria by generating 100 datasets of size m = 10,000.

	$\overline{\text{V/R}}$		$\overline{\text{S}}$		$\overline{\text{AUC}}$		$\overline{\text{pAUC}}$	
Method	Uniform	Super Uniform	Uniform	Super Uniform	Uniform	Super Uniform	Uniform	Super Uniform
std.q	0.051	0.000	43.220	4.390	0.980	0.999	0.897	0.996
prop.q(α = 0.05)	0.050	0.000	737.490	390.160	0.982	0.998	0.908	0.992
prop.q(α = cv)	0.050	0.000	848.560	458.000	0.980	0.998	0.905	0.989
IHW	0.028	0.000	110.770	69.600	0.848	0.862	0.813	0.860
BL	0.035	0.000	222.980	4.400	0.983	0.999	0.911	0.996
AdaPT	0.050	0.000	1125.680	89.960	0.971	0.886	0.882	0.907

Another key aspect of our method is the estimation of the conditional null probability $\pi_0(x) = \mathbb{P}(H_0|X = x)$. We use non-parametric estimation assisted by a covariate, motivated by the idea that genes with similar covariate values would have similar null probabilities. There are other methods in literature with the same idea, such as the method of Boca and Leek (2018) (BL), in which $\pi_0(x)$ is estimated via logistic regression. Using the estimate of $\pi_0(x)$ in BL method is possible for our proposed method. However, it is worth mentioning that, in contrast to the BL method, our proposed method also uses a non-parametric estimate of $\mathbb{P}(p_j \leq \alpha|X)$.

In this section, we conduct an additional simulation study to examine the performance of our proposed methods in the presence of covariate dependence or gene expression dependence, resulting in p-value dependence.

2.1 Extending our simulation study to include dependency among genes.

2.1.1 Dependency among genes via covariate dependence.

We consider gene expression data sets with $m = 10{,}000$ genes generated independently from normal distributions with gene-specific variance from an inverse chi-square distribution. The covariate, which affects the probability of being an EE gene, is denoted by X and assumed to follow a mixture of normal distribution. A DE gene's treatment effect is randomly generated from a normal distribution. Let j and k be the gene and treatment group indices, respectively. Let s denote a sample index within a treatment group. The sample size within a treatment group n is set to 10. Then, the data model with Y^j_{sk} as the response variable is described as follows:

$$
\begin{aligned}
&Y^j_{sk} \mid \delta^j_k, \sigma^2_j \sim N(\delta^j_k,\ \sigma^2_j),\ \text{and}\ \ \sigma^2_j \sim \text{Inv-}\chi^2_5,\ \text{where}\ j \in \{1,\dots,m\} \text{ and } s \in \{1,\dots,n\}\\
&\delta^j_0\ =\ 0\ \text{and}\ \ \delta^j_1 \mid H_j = (1-H_j)\cdot 0 + H_j \cdot N(\mu_\delta,\ \sigma^2_\delta = 0.02^2)\\
&H_j \mid X_j \sim \text{Bern}(\pi_A(X_j)), \text{ where } \pi_A(X_j) = 1 - \pi_0(X_j),\\
&X_j = W_j + a_{[j/1000]},\ \text{where}\ W_j \sim 1/2N(3, 0.5^2) + 1/2N(5, 0.5^2),\\
&a_i \sim N(0, \sigma^2_a),\ \text{where}\ i = \lfloor (j-1)/1000 \rfloor + 1 \in \{1,\dots,10\}.
\end{aligned}
\tag{1}
$$

Independence among all random variables holds except where indicated otherwise by conditioning. After generating the data set from (1), a two-sample t-test is used to obtain a p-value for testing each gene's treatment effect.

From the simulation of X_j, there are 10 different blocks of 1000 genes whose X_j are dependent, which in turn implies the dependence of the p-values within each block. The level of dependence among the p-values represents by parameter σ_a.

In specific, the simulation is conducted similarly to what is described in Section 3 of the main text, except

$$\pi_0(x) = \Phi(1.2 + \sin(5(x-4))),\ \text{where } \Phi \text{ is the cdf of the standard normal distribution}$$

and the distribution of X_j has been changed to generate dependency among p-values. The function π_0 and sample distribution of X_j is shown in Figure S1.

In this additional simulation study, our methods are compared to those described in the main text (std.q, BL,

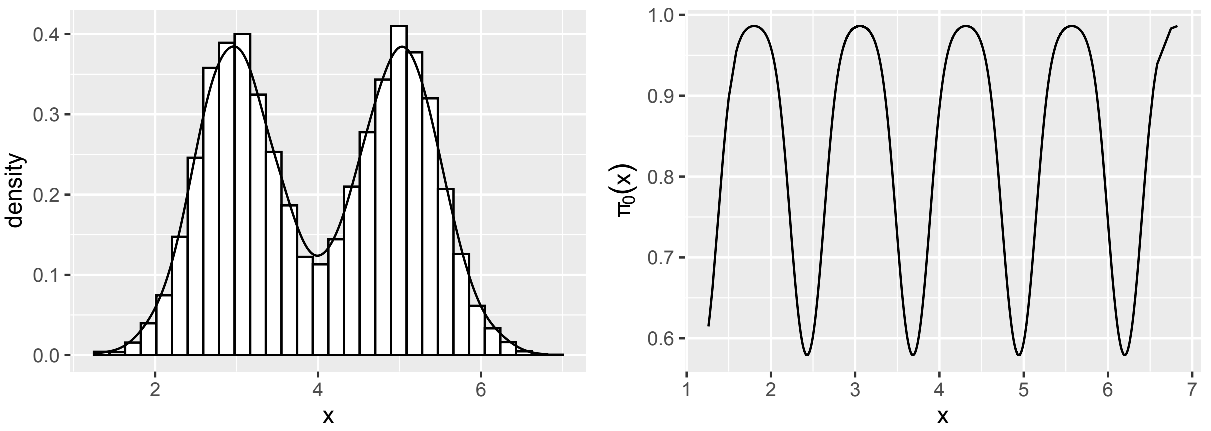

Figure S3: The left figure is the histogram of simulated covariate X for 10,000 genes, which has a bi-modal shape. The right figure is the plot of π_0 as a function of X, employed in this additional simulation studies.

AdaPT, and IHW). Our method includes `prop.q(`$\alpha = 0.05$`)`, `prop.q(`$\alpha = 0.05$`, BL)` and `prop.q(`$\alpha =$`cv)`, where `prop.q(`$\alpha = 0.05$`, BL)` indicates that only the null probability estimate in `prop.q(`$\alpha = 0.05$`)` is done with the BL approach. The methods by Lei and Fithian (2016) and Li and Barber (2019) use a specially ordered structure of hypotheses. It is not clear how they are applied to the situation where a covariate is available; therefore, we exclude these from our simulation.

We choose four values of $\mu_\delta \in \{0.15, 0.18, 0.21, 0.24\}$ and two values of $\sigma_a \in \{0.5, 1\}$. For each scenario composed of μ_δ and σ_a, we generated 100 data sets, which were used to approximate the four evaluation criteria presented in the main text.

Simulation results are shown in Figure S4. This Figure shows that our proposed method and other methods still control FDR at the nominal level 0.05. The power advantage of other methods relative to std.q tends to decrease as the strength of covariate dependency increases. Our proposed method achieves the highest power in most scenarios.

2.1.2 Dependency among genes via gene expression dependence.

To evaluate the performance of our method in different gene dependency structures, we conduct another simulation study by modifying the previous simulation (1). Here, we generate dependence at the level of gene expression, rather than through covariate values. We consider gene expression data sets with $m = 10{,}000$ genes generated from normal distributions with gene-specific variance from an inverse chi-square distribution. We

use the same notation and $\pi_0(\cdot)$ function as the previous simulation (1). Instead of the covariate dependence outlined in (1), compound symmetric correlation is assumed among genes at the observation level within each of 1000 gene groups containing 10 genes each. The data model with Y_{sk}^{j} as the response variable is described as follows:

$$
\begin{aligned}
&Y_{sk}^{j} \mid \delta_k^j, u_{sk}^i, \sigma_j^2 \sim N(\delta_k^j,\ \sigma_j^2) + \sigma_j u_{sk}^i, \text{ where } j \in \{1, \ldots m\} \text{ and } s \in \{1, \ldots n\} \\
&u_{sk}^i \sim N(0, r^2) \text{ where } i = \lfloor (j-1)/10 \rfloor + 1 \in \{1, \ldots, 1000\} \\
&\delta_0^j \;=\; 0 \text{ and } \delta_1^j \mid H_j = (1 - H_j) \cdot 0 + H_j \cdot N(\mu_\delta,\ \sigma_\delta^2 = 0.02^2) \\
&H_j \mid X_j \sim \text{Bern}(\pi_A(X_j)), \text{ where } \pi_A(X_j) = 1 - \pi_0(X_j), \\
&X_j \sim 1/2N(3, 0.5^2) + 1/2N(5, 0.5^2),\ \sigma_j^2 \sim \text{Inv-}\chi_5^2. \qquad (2)
\end{aligned}
$$

This data-generating model leads to pairwise correlation of $\frac{r^2}{1+r^2}$ between the expression levels for any two genes from the same group within each subject. We choose four values of $\mu_\delta \in \{0.15, 0.18, 0.21, 0.24\}$ and four values of $r \in \{0, 0.2, 0.4, 0.6\}$, where each r value corresponds to pairwise correlations of {0, 0.04, 0.14, 0.26} respectively. For each scenario composed of μ_δ, and r, we generated 100 data sets, which were used to approximate the four evaluation criteria presented in the main text.

Simulation results are shown in Figure S5. For the scenarios with relatively small correlations, our method performs well in this simulation study regarding FDR control and overall performance. As correlation increases, FDR levels exceed the nominal 0.05 level for all methods except AdaPT, which controls FDR but suffers from a complete loss of power. Regardless of the correlation, our method performs well in terms of AUC, and AdaPT performs well in terms of pAUC.

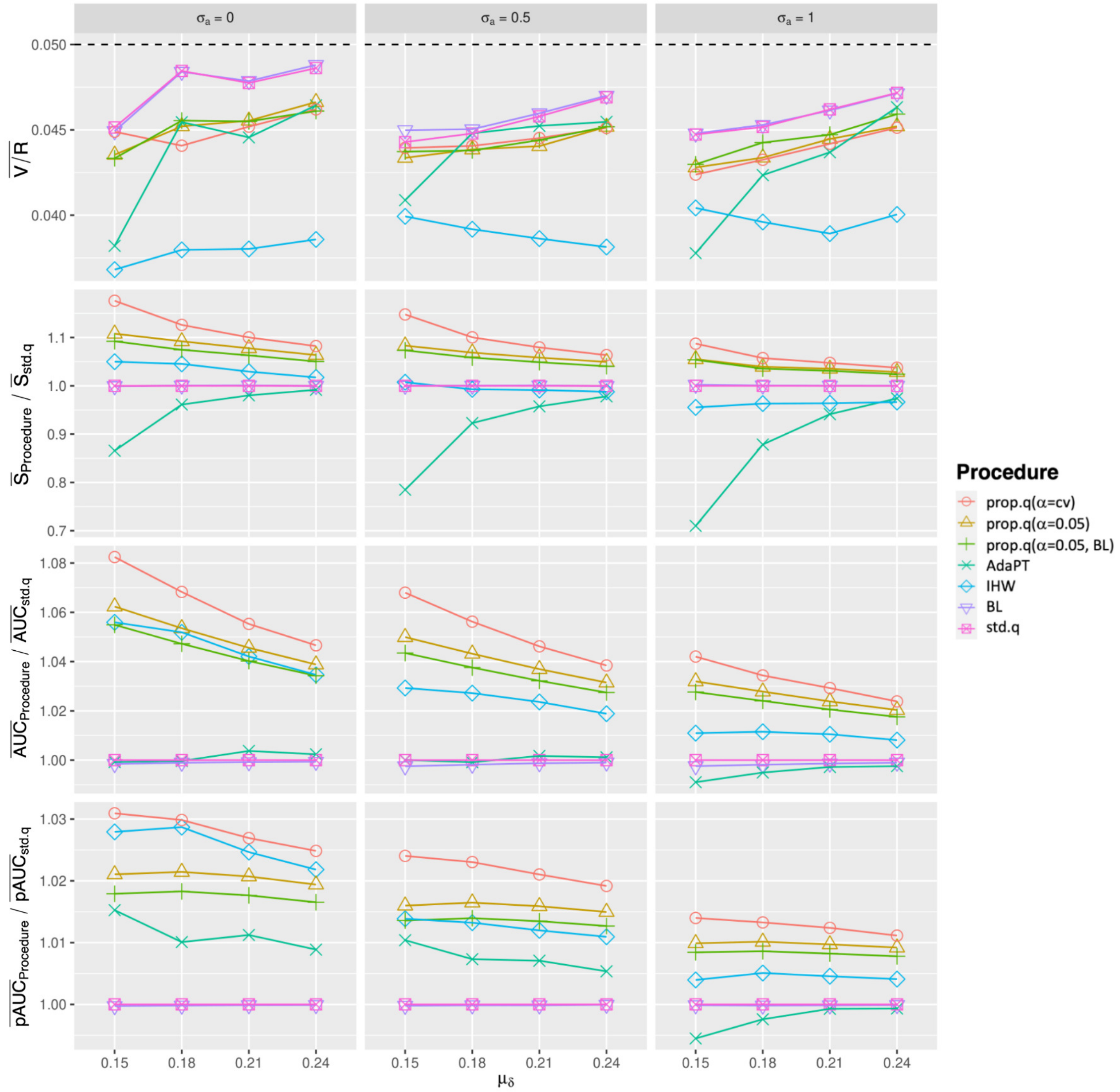

Figure S4: Simulation result with model (1) and $\pi_0(x) = \Phi(1.2 + \sin(5 * (x - 4)))$. Each column contains four graphs depicting the summary statistics of $\overline{\mathrm{V/R}}$, $\overline{\mathrm{S}}$, $\overline{\mathrm{AUC}}$, and $\overline{\mathrm{pAUC}}$, respectively.

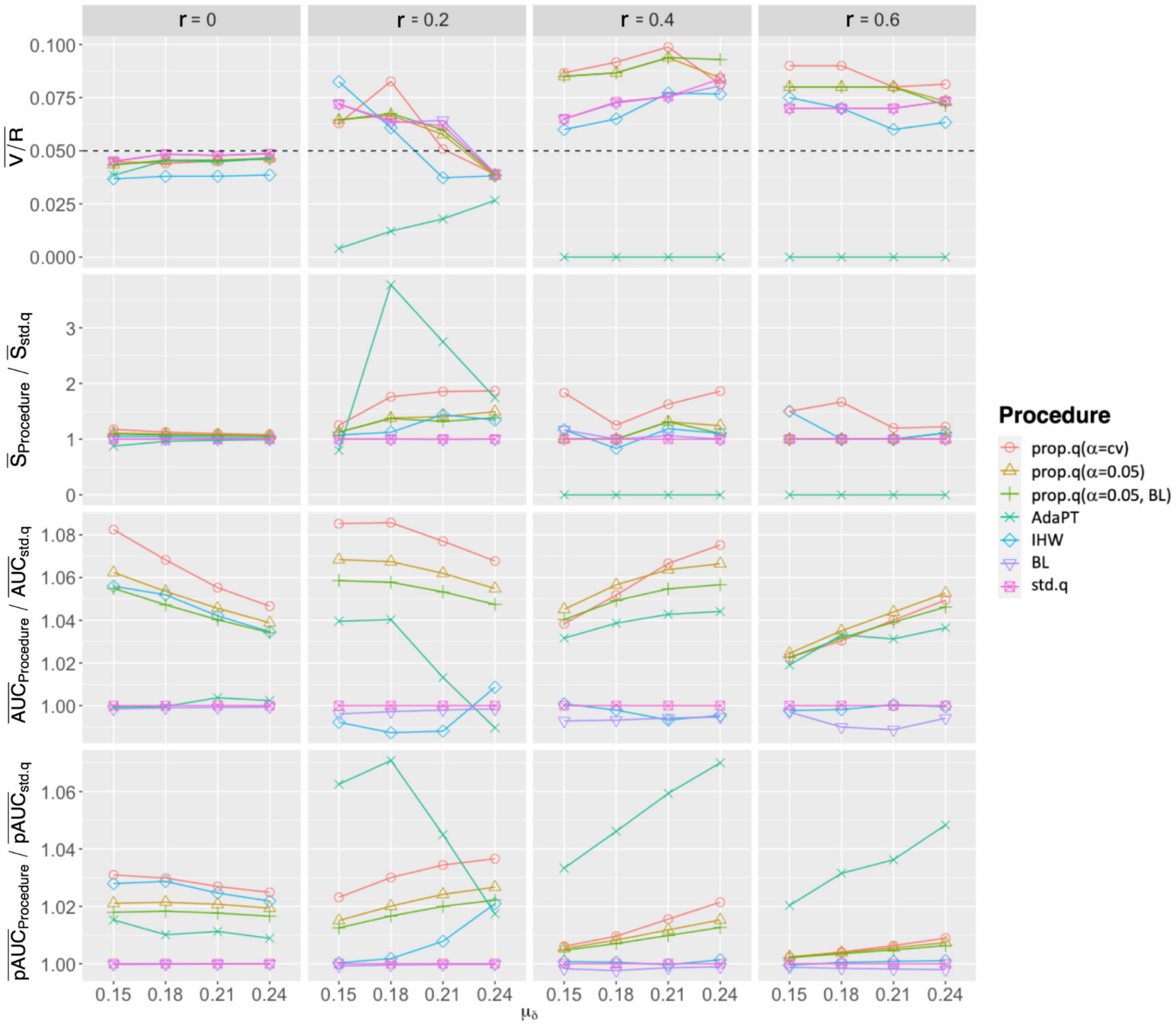

Figure S5: Simulation result with model (2) and $\pi_0(x) = \Phi(1.2 + \sin(5 * (x - 4)))$. Each column contains four graphs depicting the summary statistics of $\overline{\mathrm{V/R}}$, $\overline{\mathrm{S}}$, $\overline{\mathrm{AUC}}$, and $\overline{\mathrm{pAUC}}$, respectively.

Boca, S. M. and Leek,J.T. (2018) A direct approach to estimating false discovery rates conditional on covariates. *PeerJ*, **6**, e6035.

Lei, L. and Fithian,W. (2016) Power of ordered hypothesis testing. In Balcan, M. F. and Weinberger, K. Q. (eds), *Proceedings of the 33rd international conference on machine learning*, Proceedings of machine learning research. PMLR, New York, New York, USA, pp. 2924–2932.

Li, A. and Barber, R. F. (2019) Multiple Testing with the Structure-Adaptive Benjamini–Hochberg Algorithm. *Journal of the Royal Statistical Society Series B: Statistical Methodology*, **81**, 45–74.
